# Weight Loss and Premature Death: The 1946 British Birth Cohort Study

**DOI:** 10.1371/journal.pone.0086282

**Published:** 2014-01-21

**Authors:** Emiliano Albanese, Bjørn Heine Strand, Jack M. Guralnik, Kushang V. Patel, Diana Kuh, Rebecca Hardy

**Affiliations:** 1 Laboratory of Population Science, National Institute on Aging, Bethesda, Maryland, United States of America; 2 Norwegian Institute of Public Health, Oslo, Norway; 3 University of Maryland School of Medicine, Baltimore, Maryland, United States of America; 4 Department of Anesthesiology and Pain Medicine, University of Washington, Seattle, Washington, United States of America; 5 MRC Unit for Lifelong Health and Ageing at UCL, London, United Kingdom; Hunter College, City University of New York (CUNY), CUNY School of Public Health, United States of America

## Abstract

**Objective:**

The relationship between weight loss and mortality has important clinical and public health significance but has proved to be complex. Evidence is mixed and particularly limited on the association between weight loss in mid-life and premature death (i.e. before 65 years of age), a small albeit important segment of total mortality. We aimed to study the association between midlife weight change and mortality accounting for health and lifestyle characteristics, and also considering potential bias due to preexisting chronic diseases and smoking status.

**Design:**

Longitudinal, population-based, ‘the 1946 British’ birth cohort study.

**Subjects and Measures:**

In 2750 men and women, mortality from age 53 through 65 years was analyzed according to categories of measured 10 year weight change between 43 and 53 years. Cox's hazard ratios (HR) were progressively adjusted for socio-demographic, lifestyle and health characteristics.

**Results:**

Nearly 20% of participants lost weight and over 50% gained 5 kg or more in midlife. There were 164 deaths. Compared to those who gained between 2 and 5 kg, those who lost 5 kg or more had an increased risk of premature death independently of midlife physical activity, socio-economic circumstances and educational attainment. This association was unaltered when highest weight loss (lost more than 15 Kg) (p = 0.04) and early deaths were excluded (p<0.001), but was no longer significant after adjustment for cardiovascular risk factors and health status (HR = 1.8; 95% CI: 0.9 to 3.5).

**Conclusion:**

The inverse association between weight loss in midlife and higher risk of premature death may be explained by vascular risk factors and ill health. In consideration of the burden of premature death, closer monitoring of weight loss in mid-life is warranted.

## Introduction

Premature death (i.e. before 65 years of age) is a small segment of total mortality but of great clinical and public health importance [1]. Underweight as well as overweight and obesity are associated with an increased mortality risk in midlife populations [2]. However, higher weight and higher body mass index (BMI) seem to become protective in terms of mortality with increasing age [3], [4]. Although weight loss at older ages has been related with increased mortality rates in some studies, [5]–[8] overall evidence is conflicting [9]–[11] and the reasons underlying any such association remain unclear. The contradictory findings to date may be, at least in part, ascribed to the fallacies of observational studies, [12] and the observation that at older ages overweight may be protective with respect to some chronic diseases [13]. A number of challenges when addressing the association between weight loss and mortality have been identified which must be dealt with appropriately in analyses. First, smoking is a strong risk factor for mortality but smokers also tend to have a lower body weight than non-smokers. Second, potentially mediating vascular risk factors may improve with weight loss. Finally, there is the possibility that health status and clinical conditions which lead to mortality also cause weight loss either through the clinical disease process or by prompting attempts to lose weight after a condition is diagnosed [14]. Weight loss is also a marker of disease severity, [15] and may aggravate the vital prognosis of several diseases [16]. The association of weight change with mortality has not yet been studied addressing comprehensively these challenges that might bias the findings, [17] and there is a lack of evidence on the association between weight loss in midlife, as opposed to weight loss in older age, and premature death.

We used data from a British birth cohort study to test the hypothesis that weight loss over a 10-year period in midlife (43–53 years) would be associated with an increased risk of all-cause premature death between 53 and 65 years. We also investigated whether health conditions (including cancer history, as well as cardiovascular diseases and risk factors), smoking, and body size at age 43 explained any observed associations.

## Materials and Methods

### Ethics statement

Participants provided written informed consent and the Multicentre Research Ethics Committee (MREC) approved the study.

### Data Availability

Bona fide researchers can apply to access the NSHD data via a standard application procedure (see details at: http://www.nshd.mrc.ac.uk/data.aspx).

### Study population and follow-up

The MRC National Survey of Health and Development (NSHD) is an ongoing birth cohort study of a socially-stratified sample of 5 362 newborns born in England, Scotland and Wales in one week in March 1946, and followed up 24 times. Height and weight were measured at all study waves to the nearest 0.1 kg with participants in light clothing and no shoes (self-reported at 20 and 26 years). In 1989 and 1999 trained nurses used standardized protocols at home visits to assess health and lifestyle characteristics. Cohort members were flagged for death on the National Health Service (NHS) Central Register. The start of mortality follow-up was taken as 1999 when cohort members were aged 53 years and ended in March 2011, just before the study members' 65th birthday. Of the 5 362 cohort members 469 had died before age 53 years, 640 had withdrawn from the study, 580 lived abroad, and 638 were untraced or non-responders. Of the remaining 3 035, we excluded those who were not weighed and measured at both 43 and 53 years (n = 274) and those who did not have mortality data available (n = 11) leaving 2 750 for analysis. A further 182 were excluded from complete-case analyses due to missing covariate information.

### Exposure and covariates assessment

The exposure variable was 10 year weight change calculated by subtracting weight at age 43 from weight at age 53 years. We derived a categorized variable for weight change using the following groups: [5] 1) −5 kg or more; 2) –4.99 to–2 kg; 3) −1.99 to +2 kg; 4) +2 to +5 kg; 5) 5.01 kg or more.

We classified educational attainment achieved by the age of 26 years according to the Burnham scale into: [18] 1) higher education/postsecondary college or university degree, 2) advanced secondary education/university entrance qualification, 3) ordinary secondary education/junior high school qualifications, 4) below ordinary secondary qualification, and 5) no qualification. Other covariates were included consistent with previous findings from the NSHD. Household occupational social class at age 53 years according to the Registrar General system: 1) I professional and II intermediate, 2) III non-manual skilled, 3) III manual skilled, 4) IV partly skilled and V unskilled; [19] smoking habit at 43 and 53 years: 1) no smokers at both ages, 2) smokers at 43 or at 53 years, 3) smokers at both ages. Additional potential confounders related to health status included: [10] physical activity level at 43 and 53 years (defined as any sports, exercises or vigorous leisure activities taken in the month preceding the: none, 1–4 times a month or more than 4 times a month); Rose angina Grade I or II elicited by standardized questions; self-reported stroke; diagnosed or undiagnosed hypertension at 43 years (those on antihypertensive treatment and/or with measured systolic blood pressure > = 160 and diastolic > = 100); lung function at 43 years (forced expiratory volume (FEV1) measured using the Micromedical turbine electronic spirometer). These covariates capture participants' morbidity status and are also potentially associated with dieting and changes and/or restrictions in physical activity level and are therefore best measured at the beginning of the weight change period. Health characteristics at start of mortality follow-up were determined in 1999 (at 53 years) by eliciting information about history of type II diabetes, cancer, liver disease and thyroid disorder between 43 and 53 years and by recording any recent (past 3 weeks) abdominal or chest surgery and finally by asking the question: ‘have you recently felt that you are ill?’ (y/n) to measure self-rated health at 53 years. This latter set of health characteristics likely impacts both the 10 year weight change period and the follow-up period.

### Statistical analysis

We compared participants' characteristics across weight change categories using ANOVA and χ^2^. *A priori* power calculations (at 90% and 5% significance) confirmed that a hazard ratio (HR) of 1.03 per kg of weight change could be detected assuming a linear relationship. Weight change predicted mortality similarly in men and women (P-value for sex interaction  = 0.93), so results are presented for men and women together adjusted for sex.

Survival across weight change categories was first assessed by graphical inspection of Kaplan-Meier plot, and a formal test using Schoenfeld residuals showed no violation of the proportionality assumption (P = 0.41). To assess the relationship between weight change and mortality we performed two separate analyses. First, we included weight change as a continuous variable and used restricted cubic spline with three knots to assess the shape of the association with mortality rates. The association with mortality was assessed using the Cox proportional hazards model adjusting for sex and weight at 43 years. The p-value for overall significance of the association was obtained comparing the spline models with the null model using a Wald test (N = 2 750, deaths  = 164). Second, the relationship between weight change and mortality was modeled using the Cox model with weight change in categories (above), initially on the maximum sample with weight change available (n = 2 750, deaths  = 144). The unadjusted model was then refitted on a reduced sample with complete data on all covariates (n = 2 568) to assess potential bias introduced by missing data. Adjustment was then made for socio-demographic, lifestyle and health characteristics in stages to assess confounding. To account for the possibility that existing underlying disease, which results in mortality, may have caused the weight loss, [17] we carried out sensitivity analyses excluding individuals with highest weight loss (15 Kg or more; n = 20), and, separately, deaths that occurred in the first 2 years of follow-up. We then included interaction terms of weight change by obesity (BMI >30 Kg/m^2^) at 43 years, and by smoking status (yes/no) at the beginning of the weight loss period (43 years) to formally test whether obesity and smoking status modified the association of weight change with mortality. We also conducted separate analyses by subgroup where evidence of an interaction was observed. Finally, because weight change is likely related to body size, in additional models we investigated the relationship between BMI (in categories; underweight: BMI <18.5, normal weight: 18.5< BMI <25, overweight: 25< BMI <30, and obesity: BMI ≥30 Kg/m^2^) at 43 years and mortality controlling for sex, and weight change. A similar model was fitted replacing BMI at 43 years with BMI at 53 years. We conducted the statistical analyses using STATA software, version 12.0 (STATA Corp. – College Station, Texas).

## Results

Similar percentages of men and women experienced weight loss, and 11% lost more than 2 kg and 48% gained more than 5 kg between 43 and 53 years. Participants grouped by weight change categories differed across a number of factors (table 1). For instance, there were greater percentages of men, those in lower social classes, smokers and physically inactive in the groups who lost weight compared with others. Weight gainers were lighter at 43 years than those who lost weight but heavier at 53 years. Cardio-vascular disease at 43 years (angina, stroke and hypertension) and prevalent diseases at 53 years were uncommon and there was little difference across groups, though lung function was lower in those who lost compared to those who gained weight (table 1).

**Table 1:** Characteristics of the analytic sample (n = 2568) by categories of 10-years weight change, the 1946 British birth cohort study.

	Weight change between 43 and 53y					
	−5 Kg or more	−4.9 to −2 kg	−2.1 to 2 Kg	2.1 to 5 Kg	5.1 Kg or more	
**Sample size**	125 (4.9)	168 (6.5)	500 (19.5)	540 (21.1)	1235 (48.1)	
**Sex, %**						0.007
Women	48.0	47.1	48.2	46.9	54.8	
Men	52.0	52.9	51.8	53.1	45.2	
**Education, %**						0.115
Higher	5.6	9.5	10.0	11.3	9.15	
Advanced secondary	17.6	17.3	24.0	27.8	25.8	
Ordinary secondary	30.4	22.1	24.4	21.7	24.2	
Vocational	42.4	44.6	37.2	34.6	36.0	
No qualification	4.0	6.6	4.4	4.6	4.8	
**SEP, %**						<0.001
I & II	28.0	31.5	43.8	48.5	43.9	
III non-manual	20.8	19.1	21.0	21.3	25.5	
III manual	29.6	22.6	18.8	18.3	15.1	
IV & V	21.6	26.8	16.4	11.8	15.6	
**Smoking habit, %**						<0.001
No smoker	55.2	63.1	68.4	74.6	69.0	
Smoke once (either 43 or 53y)	12.0	7.1	8.0	7.4	14.2	
Smoker	32.8	29.7	23.6	18.0	16.8	
**Physical act. level at 43y, %**						0.003
None	67.2	51.2	50.8	44.6	51.9	
1–4 times a month	9.6	13.1	15.2	17.0	15.2	
4+ times a month	23.2	35.2	34.0	38.0	32.8	
**Physical act. level at 53y, %**						0.024
None	60.0	52.4	46.8	44.7	49.4	
1–4 times a month	10.4	17.7	16.6	18.3	18.8	
4+ times a month	29.6	30.4	36.6	37.4	31.7	
**Health at 43years, %**						
Angina	0.8	3.0	1.2	1.9	1.5	0.482
Stroke	1.6	0.6	0.2	0.2	0.2	0.102
Hypertension	10.4	6.0	7.2	5.4	5.5	0.172
Lung function FEV_1_, mean (sd)	2.85 (0.8)	2.89 (0.7)	3.00 (0.7)	3.05 (0.7)	3.02 (0.7)	0.014
**Health at 53years, %**						
Recently felt ill	46.4	37.5	34.2	30.9	36.0	0.016
Diabetes	8.0	6.5	4.0	1.5	1.7	<0.001
Had cancer in the past 10y	4.0	4.2	2.4	2.0	3.6	0.1316
Thyroid disorders in the past 10y	6.4	4.2	5.4	2.6	4.7	0.148
Liver disease in the past 10y	2.4	2.4	0.6	0.2	1.1	0.029
Had recent major surgery	3.2	0.6	0.2	0.9	0.6	0.013
**Weight (Kg) at 43y, mean (sd)**	81.4 (16.9)	74.6 (14.6)	71.0 (12.9)	70.6 (12.6)	72.6 (13.6)	<0.001
**Weight (Kg) at 53y, mean (sd)**	71.1 (15.4)	71.3 (14.6)	71.4 (12.8)	74.2 (12.6)	82.8 (15.0)	<0.001
**BMI (Kg/m^2^) at 43y, mean (sd)**	29.0 (6.2)	26.0 (4.4)	24.9 (3.7)	24.6 (3.5)	25.5 (4.1)	<0.001
**BMI (Kg/m^2^) at 53y, mean (sd)**	25.5 (5.6)	25.2 (4.6)	25.3 (3.8)	26.1 (3.5)	29.4 (4.8)	<0.001

P values for the difference across weight change categories calculated with ANOVA (for continuous variables) and χ^2^ tests (for categorical variables).

### Weight Change and Mortality

**Figure 1:**
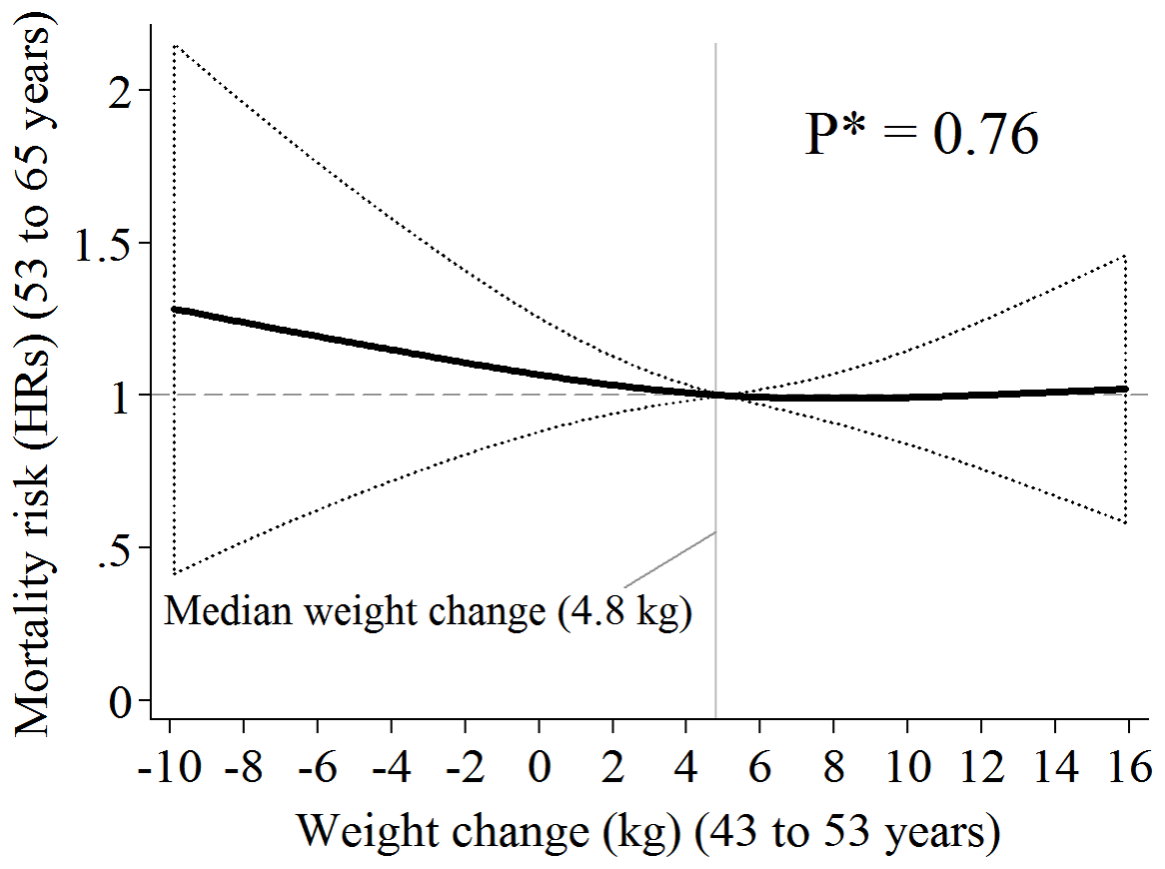
Weight Change (kg) Between 43 and 53 Years and all-Cause Mortality Hazard Ratios (HRs) Between 53 and 65 Years (95% CI bands, dotted lines), in the 1946 British Birth Cohort (n = 2 568; deaths  = 141). HRs are from Fully Adjusted* Cox Regression Using Cubic Splines and Median Weight Change ( = 4.8 Kg) as Reference Point.

The analysis using spline models suggested that the association between weight change and mortality rates was not linear (figure 1). There was increasing hazard of mortality with increasing weight loss, but no increase for weight gain. Mortality risks by weight change categories are reported in table 2, the highest weight loss group has a particularly high rate compared with all other groups. In unadjusted models, compared to modest weight gain (2 to 5 kg), highest weight loss (−5 kg or more) in midlife was associated with up to nearly a four folds increase in mortality rate up until 65 years in both the full and complete-case sample. Adjustment for weight at 43 years and sex (model 2) slightly attenuated this estimate, with further attenuation after adjustment for physical activity level at 43 and 53 years, education and SEP (model 3). The HR for the greatest weight loss group was reduced further and was no longer significant when also adjusted for cardio-vascular disease at 43 years and health status at 53 years (model 4).

**Table 2:** Association between 10-year (43 to 53 years) weight change categories and all-cause premature mortality (53 to 65 years) in the 1946 British birth cohort.

	N	No. of Deaths^1^	Follow-up^2^	Rates^3^	Full Sample^1^	Reduced sample^4^			
**Weight change**						**Model 1**	**Model 2**	**Model 3**	**Model 4**
					**HR (95% CI)**	**HR (95% CI)**	**HR (95% CI)**	**HR (95% CI)**	**HR (95% CI)**
−5 kg or more	141	22	1543	14.3	3.8 (2.1, 6.7)	3.8 (2.0, 7.0)	3.3 (1.7, 6.2)	2.8 (1.4, 5.3)	1.8 (0.9, 3.5)
−5 to −2 kg	179	13	2013	6.5	1.7 (0.9, 3.3)	1.8 (0.9, 3.6)	1.7 (0.8, 3.4)	1.5 (0.7, 3.1)	1.2 (0.6, 2.5)
−2 to +2 kg	534	36	6173	5.8	1.6 (0.9, 2.6)	1.6 (0.9, 2.7)	1.6 (0.9, 2.8)	1.5 (0.9, 2.6)	1.3 (0.8, 2.3)
2 to 5 kg	570	25	6657	3.8	1 (Reference)	1 (Reference)	1 (Reference)	1 (Reference)	1 (Reference)
+5 kg or more	1,326	68	15408	4.4	1.2 (0.7, 1.9)	1.2 (0.7, 2.0)	1.2 (0.7, 2.0)	1.2 (0.7, 1.9)	1.2 (0.7, 2.0)

Abbreviations: CI, confidence intervals; HR, hazard ratio;

^1^ Based on the sample of participants with available data for mortality and weight change (n = 2 750; deaths  = 164).

^2^ Follow-up in person-years.

^3^ Mortality rates are percentages per 1,000 person-years.

^4^ Sample of participants with available data for mortality, weight change and all covariates through model 4 (n = 2 568; deaths = 144).

Model 1– unadjusted.

Model 2– adjusted for weight at age 43 years and sex.

Model 3– as in model 2 plus: educational attainment, socio-economic position, exercise level at 43 and at 53 years.

Model 4– as in model 3 plus: cardio-vascular disease and risk factors at 43y (angina, stroke, lung function (FEV_1_), hypertension), health conditions at 53y (cancer and diabetes history, thyroid and liver disorders, recent major surgery or illness) and smoking habits at 43 and 53y.

In sensitivity analyses, when participants who lost more than 15 kg were excluded (n = 20), the HR for the highest weight loss category (−5 to <−15 Kg) compared with the reference group was only modestly attenuated in a model adjusted for sex and body weight at age 43 years (HR = 2.5; 95% CI: 1.3, 5.0) when compared with the main analysis. Similarly, when early deaths (before age 55 years) (n = 12) were excluded, the HR for the high weight loss group was significant through to model 3 (HR = 1.8; 95% CI 1.0, 3.4). Also consistent with the main analysis, the HR was considerably attenuated and not significant in the fully adjusted model (HR = 1.4; 95% CI 0.7, 2.7).

In stratified analyses, the increased mortality rate in the highest weight loss group (compared to the reference) was stronger and remained significant after full adjustment (model 4) in non-smokers (HR = 2.9; 95% CI: 1.00, 6.7) but not in smokers at age 43 years (HR = 1.42; 95% CI: 0.5, 3.8) (p value for smoking by weight change interaction in model 4 = 0.09). There was no evidence of an interaction between weight change and obesity status (BMI>30 kg/m2) at 43 years (p = 0.50). Finally, we found that participants who were obese at 43 years (n = 334; 12% of the study sample) had a higher mortality risk (HR = 1.7; 95% CI: 1.1, 2.7) compared to those who were in the normal weight category (18.5<BMI<25; n = 1 421) at the same age. This association was independent of their weight change between 43 and 53 years and was hardly attenuated by adjustment for all other covariates. Conversely, those who were obese at 53 years (n = 666; 24% of the study sample) did not have a significantly higher mortality risk (HR = 1.4; 95% CI: 0.9, 2.7) compared to those who were normal weight (n = 902) in similar models.

## Discussion

In this prospective birth cohort study we found that compared to modest weight gain (less than 5kg), weight loss of more than 5 kg in mid-life was associated with a higher risk of mortality before old age (i.e., 65 years). The association was strongly attenuated when cardiovascular risk factors and health status were taken into account.

The obesity epidemic is spreading worldwide [20] and its link with morbidity and mortality is well documented [21], [22]. A few years ago, Sorensen suggested that the fact that mortality is increased by weight loss is a ‘painful paradox’ that clinicians may regard with disbelief and some researchers have ascribed to the fallacies of observational studies [12]. Our findings are in agreement with previous research and suggest that smoking, and underlying diseases that precede or coexist with weight loss may confound the association with mortality [17]. Interestingly, our results were unaltered when we conducted sensitivity analyses excluding highest weight loss or early deaths intended to address potential confounding due to pre-existing disease as others have done [23]. Furthermore, our stratified analyses by smoking status showed that in smokers the relationship between weight loss and mortality was more largely accounted for by ill health, than in non-smokers. A possible explanation is that health is poorer in smokers compared to non-smokers, and initial symptoms and clinical signs of disease may be milder in the latter, and thus they might have been less captured by our health measures.

Our finding that health state strongly attenuates the association between weight loss of more than 5kg and mortality is consistent with a previous study also conducted in a UK population-based sample [24]. However, weight loss remained significantly associated with higher mortality risk in studies that excluded from analysis participants with poor health at baseline [25]. Both positive [26]–[28] and null [24], [29] associations have been reported between weight loss and mortality. Discrepant results may be in part explained by age differences at both baseline and follow-up among studies, by cohort effects in mortality risk, [22] or how health status was measured and accounted for in the analyses [17]. Unlike previous studies that were typically conducted in the elderly, we focused on midlife weight change and premature death (i.e. <65) [12], [23]. To our knowledge this is the first study to do this and to show an association of this early weight loss with mortality [10], [30]. In addition, traditional methods of accounting for ‘reverse causality’, like removing early deaths and controlling for smoking, did not account for the effect of weight change on mortality risk, different from health characteristics, which should be accounted for in future studies.

### Strengths and limitations

Our study has limitations. Mortality risks were higher amongst those who were not compared to those who were included in our analysis [31]. However, our analytic sample (n = 2 568) comprised 84% of those who took part in the 1989 resurvey (baseline of the weight loss period). Moreover, response rates in the available sample in 1989 and 1999 were high (80% or more) and the NSHD study sample broadly maintained its nationwide representativeness through the follow-up in 1999 [32]. We focused on premature mortality, a small (albeit important) segment of total mortality and indeed the total number of deaths was small (n = 164). Consequently, causes of deaths could not be considered because of low statistical power, and some estimates in the current analyses were imprecise. We were not able to distinguish between intentional and unintentional weight loss. However, if those who intentionally lost weight in our sample did so to improve their health they might have lowered their mortality risk hence shifting our findings towards the null [6]. The beneficial effect on health of weight loss in overweight and obese subjects is plausibly due to loss of fat mass, [33] that we were unable to distinguish between fat and lean mass loss is therefore another limitation of our study. Comparisons were not made with those who maintained their weight in midlife (i.e. −2 to 2 kg category), who might be assumed to have lowest mortality risks, but with those who gained between 2 and 5 kg. However, the 2 to 5 kg weight change group and (accordingly) the median weight change (+4.8 Kg) as referents in our analyses were chosen consistently with studies that reported lowest mortality risks in moderate weight gainers [34]. Strengths of the study include the birth cohort design, the sample representativeness and the available data on cardio-vascular disease and risk factors before weight change and health characteristics after weight change occurred; the latter being unlikely to improve with, or result from, previous weight change (cancer, diabetes, thyroid and liver disorders and major surgery). Finally, we carried out sensitivity analyses to assess whether results were influenced by reverse-causality, and stratified by obesity and smoking status to assess possible effect modification.

Overall our findings may reflect illness-related weight loss, [9] and also the aggravating effects of weight loss on the vital prognosis of those with certain medical conditions [16]. This is somewhat consistent with the conclusions of a recent systematic review that questioned the health benefits of weight loss [10]. Indeed, weight loss is currently not recommended in geriatric patients, [1] and the media have alluded that ‘chubby may be the new healthy’ [35].

## Conclusions

In the present study the direct association between weight loss and risk of premature death was largely explained by modifiable risk factors before and treatable clinical conditions after weight loss occurred, and was independent of obesity at 43 years. Together this may suggest that weight loss in midlife as well as obesity warrants monitoring to improve prevention and tailor treatment.
